# Differences in Lipid Profiles and Atherogenic Indices Between Hypertensive and Normotensive Populations: A Cross-Sectional Study of 11 Chinese Cities

**DOI:** 10.3389/fcvm.2022.887067

**Published:** 2022-05-17

**Authors:** Wenke Cheng, Lili Wang, Siwei Chen

**Affiliations:** ^1^Medical Faculty, University of Leipzig, Leipzig, Germany; ^2^Retirement Clinic, Tengzhou Central People's Hospital, Shandong, China; ^3^Geriatric Medicine Clinic, Tengzhou Central People's Hospital, Shandong, China; ^4^Department of Cardiovascular Medicine, The Third Hospital of Nanchang, Jiangxi, China

**Keywords:** lipid, triglycerides, high-density lipoprotein cholesterol, low density lipoprotein, total cholesterol, hypertension, Bayesian network model

## Abstract

**Background:**

Several previous studies have reported that dyslipidemia is associated with the risk of hypertension, but these studies are mainly conducted in European and US populations, with a very few studies in the Asian population. Moreover, the effects of atherosclerotic indices, including atherogenic coefficient (AC) and atherogenic risk of plasma (AIP), on hypertension in Asians have not been well described so far.

**Methods:**

From 2010 to 2016, altogether 211,833 Chinese adults were ultimately recruited at the health centers in 11 Chinese cities (including Shanghai, Beijing, Nanjing, Suzhou, Shenzhen, Changzhou, Chengdu, Guangzhou, Hefei, Wuhan, and Nantong). Differences in continuous variables between the two groups were analyzed by the Mann–Whitney test, while those in categorical variables were examined by the Chi-squared test. Logistic regression was applied to evaluate the association between lipid profiles and the risk of hypertension. The predictive values of AC and AIP for the incidence of hypertension were analyzed using the area under the receiver operating characteristic (ROC) curve. Meanwhile, Bayesian network (BN) models were performed to further analyze the associations between the different covariates and the incidence of hypertension.

**Results:**

A total of 117,056 participants were included in the final analysis. There were significant differences in baseline characteristics between normotension and hypertension groups (*p* < 0.001). In multivariate logistic regression, the risk of hypertension increased by 0.2% (1.002 [1.001–1.003]), 0.2% (1.002 [1.001–1.003]), and 0.2% (1.002 [1.001–1.003]) per 1 mg/dl increase in total cholesterol (TC), low-density lipoprotein (LDL), and non-high-density lipoprotein cholesterol (non-HDL-c), respectively. However, after adjusting for body mass index (BMI), an increase in HDL level was associated with a higher risk of hypertension (*p* for a trend < 0.001), and the risk of hypertension increased by 0.6% per 1 mg/dl increase in HDL-c (1.006 [1.003–1.008]). In women, AC had the highest predictive value for the incidence of hypertension with an area under the curve (AUC) of 0.667 [95% confidence interval (CI): 0.659–0.674]. BN models suggested that TC and LDL were more closely related to the incidence of hypertension.

**Conclusions:**

Overall, lipid profiles were significantly abnormal in the hypertensive population than in the normotensive population. TC and LDL were strongly associated with the incidence of hypertension. TC, LDL, and non-HDL-c levels show a positive association, HDL-c shows a negative association, while TG is not significantly associated with the risk of hypertension. After adjusting for BMI, HDL-c turns out to be positively associated with the risk of hypertension. In addition, AC has a good predictive value for the incidence of hypertension in women.

## Introduction

Hypertension is not only one of the most prevalent public health problems in the world, but also a major contributor to the global burden of disease and death (1). It is clinically characterized by prolonged, sustained elevation of blood pressure (2). The overall prevalence of hypertension is estimated to be 29.2% by 2025 (3), in other words, there will be 1.56 billion people with hypertension, having one-third of the hypertension population in China (4, 5).

Generally, dyslipidemia consists of the elevated total cholesterol (TC) or low-density lipoprotein cholesterol (LDL-c), or elevated triglycerides (TG), or decreased high-density lipoprotein cholesterol (HDL-c).

Dyslipidemia and hypertension are two different diseases, but both of them may induce the risk of cardiovascular diseases (CVDs) and commonly occur synergistically in the affected individuals (6). Studies have suggested that patients with dyslipidemia and hypertension are associated with a significantly higher risk of cardiovascular mortality than the pooled risk caused by dyslipidemia and hypertension separately (7, 8). In this regard, such a synergistic effect is crucial to elucidate the relationship between dyslipidemia and hypertension. As reported in several previous studies, dyslipidemia is associated with the risk of hypertension, but these studies are conducted mainly in European and US populations (9–11), while a few of them focus on the Asian population. Moreover, the effects of atherosclerotic indices, including atherogenic coefficient (AC) and atherogenic risk of plasma (AIP), on hypertension in Asians have not been well described so far. Therefore, the present cross-sectional study based on over 100,000 Chinese was conducted to compare the lipid differences and atherosclerotic indices between hypertensive and normotensive populations and to further determine the relationship between lipid levels and atherosclerotic indices and the incidence of hypertension.

## Methods

### Study Design and Data Extraction

This was a cross-sectional study performed based on the DATADRYAD database (www.Datadryad.org), a computerized database established by the Rich Healthcare Group of China. The original data were provided by Chen et al. (12). The raw data were downloaded from this site free of charge. Specifically, the original study aimed to assess the associations of body mass index (BMI) and age with the incidence of diabetes in Chinese adults. From 2010 to 2016, altogether 211,833 Chinese adults were ultimately recruited from the healthcare centers in 11 Chinese cities (namely, Shanghai, Beijing, Nanjing, Suzhou, Shenzhen, Changzhou, Chengdu, Guangzhou, Hefei, Wuhan, and Nantong). All participants completed a detailed questionnaire assessing demographics, lifestyle, and family history of chronic disease during their initial visit to a healthcare center. Baseline information, including clinical and biochemical measurements, was recorded. Clinical measurements, including weight, height, and blood pressure, were taken by the trained staff. The following biochemical parameters were measured, including TC, HDL-c, LDL-c, serum creatinine (Scr), blood urea nitrogen (BUN), alanine aminotransferase (ALT), and aspartate aminotransferase (AST). BMI was equal to the weight divided by height squared. All data were collected under standardized conditions and performed following the uniform procedures.

As Chen et al. waived all copyrights and related ownership of raw data, these data could be used for secondary analyses without infringing on authors' rights. In addition, the original study was approved and informed consent was waived by the Rich Healthcare Group Review Board, and baseline information was retrieved retrospectively (13). This study was in compliance with the Declaration of Helsinki.

### Study Population

**Figure 1:**
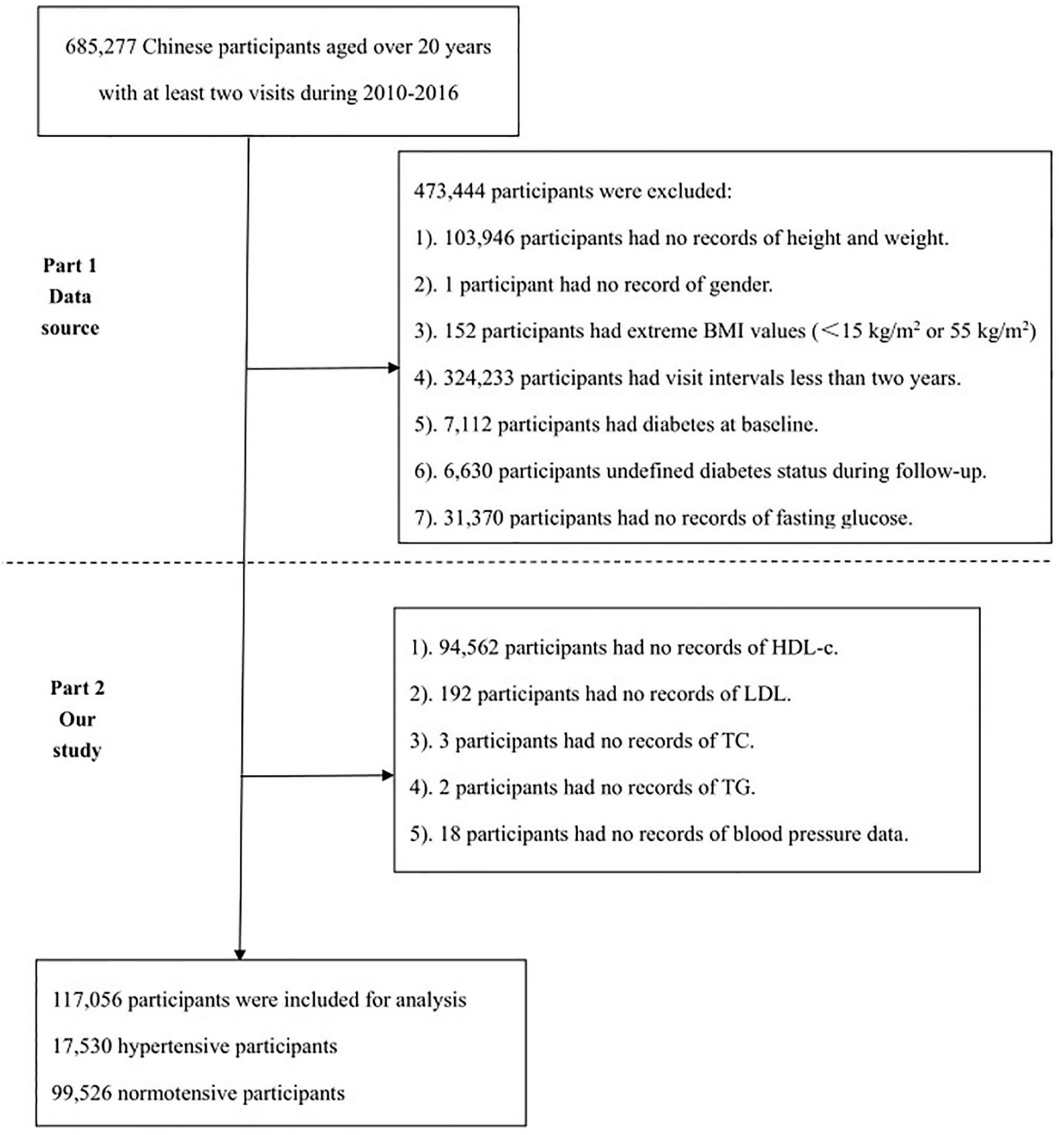
The flowchart of the study. The first part is the flowchart of the original study and the second part is the flowchart of the current study.

Initially, 685,277 Chinese adults who were aged over 20 years were recruited from 32 health screening centers in 11 cities in China from 2010 to 2016. As shown in Figure 1, the participant selection process consisted of two parts. The first part was the flowchart of the original study, which included 211,833 participants, where the reasons for their exclusion were as follows: (1) participants with no records of height or weight (*n* = 103,946); (2) participants with no records of gender (*n* = 1); (3) participants with extreme BMI values (<15 kg/m^2^ or 55 kg/m^2^) (*n* = 152); (4) participants whose visit intervals were less than 2 years (*n* = 334.233); (5) participants with diabetes at baseline (*n* = 7,112); (6) participants with undefined diabetes status during a follow-up (*n* = 6,630); and (7) participants with no records of fasting glucose (*n* = 31,370). The second part is the flow chart of the current study, with the following reasons for their exclusion: (1) participants with no records of HDL-c (*n* = 94,562); (2) participants with no records of low-density lipoprotein (LDL) (*n* = 192); (3) participants with no records of TC (*n* = 3); (4) participants with no records of TG (*n* = 2); and (5) participants with no records of blood pressure data (*n* = 18). Ultimately, a total of 117,056 participants, including 17,530 hypertensive and 99,526 normotensive participants, were participated in this study for further analysis.

### Exposures of Interest and Outcome Measures

The exposures of interest were plasma lipid profiles, including TC, TG, LDL, HDL-c, and non-HDL-c, and atherosclerotic indices, including AC and AIP. AC, AIP, and non-HDL-c were calculated using the following formulas (14): AC = (TC-HDL-c)/HDL-c; AIP = Log (TG/HDL-c); and non-HDL = TC- HDL-c. With reference to the Chinese guidelines for the prevention and treatment of hypertension (2018 version) (15), participants were divided into hypertensive and normotensive groups according to their baseline blood pressure levels. Hypertension was defined as systolic blood pressure (SBP) ≥140 mmHg or diastolic blood pressure (DBP) ≥ 90 mmHg. Moreover, hypertension was further subdivided into mild hypertension (Grade I; SBP 140–159 mmHg or DBP 90–99 mmHg), moderate hypertension (Grade II; SBP 160–179 mmHg or DPB 100–109 mmHg); and severe hypertension (Grade III; SBP ≥ 180 mmHg or DBP ≥ 110 mmHg). Normotension was defined as SBP < 140 mmHg and DBP < 90 mmHg.

The primary outcome of this paper was to assess the differences in lipid profiles, AC, and AIP between hypertensive and normotensive groups, and the association of different lipid levels and combinations with the incidence of hypertension. The secondary outcome was to construct a Bayesian model to assess the potential relationships between baseline characteristics and the incidence of hypertension. Moreover, the importance of lipid levels, AC, and AIP for the incidence of hypertension was assessed.

### Statistical Analyses

All continuous variables with a skewed distribution were expressed as median and interquartile range (IQR), whereas categorical variables were expressed as percentages. For continuous variables, differences between the two groups were analyzed using the Mann–Whitney test, while differences in categorical variables were examined using the Chi-squared test. According to blood pressure levels, the total population was divided into four groups, including Normal, Grade I, Grade II, and Grade III. Then, multiple comparisons of lipid levels, AC, and AIP were performed by Kruskal–Wallis one-way analysis of variance (ANOVA) with Dunn's test. Subgroup analyses stratified by age (<60 years, ≥60 years), gender (male, female), BMI (<23 kg/m^2^, ≥23 kg/m^2^), ALT (<40 U/L, ≥40 U/L), and AST (<40 U/L, ≥40 U/L) were performed. In addition, TC, TG, LDL, HDL-c, non-HDL-c, AC, and AIP were categorized into quartiles. A *p* for trend was employed to assess the trend of associations between lipid profiles, AC, and AIP and the incidence of hypertension. The predictive values of AC and AIP for the incidence of hypertension were analyzed using the area under the receiver operating characteristic (ROC) curve. Moreover, age, gender, BMI, fasting plasma glucose (FPG), ALT, AST, BUN, Scr, smoking status, drinking status, and family history of diabetes were adjusted for regression analyses. Statistical analyses were conducted by SPSS 26.0 and GraphPad 9.0.

### Bayesian Network

The Bayesian network (BN) model is one of the probabilistic graphical models, which combines probability theory and graph theory to reveal the probabilistic dependencies between variables (nodes). In short, it is about inferring the probability of occurrence of an outcome in the presence of multiple conditional variables. Firstly, in the SPSS Modeler (version 18.0) model section, the BN model is constructed based on the tree augmented native (TAN) algorithm, and the parameter learning method is selected as a Bayesian adjustment for small cell counts (16). The arrow connecting two nodes indicates that the two random variables are causally or unconditionally independent; if there is no arrow connecting two nodes, it indicates that the random variables are conditionally independent (17). Meanwhile, the importance of the variables is obtained.

## Results

Demographic data and baseline characteristics of eligible participants are displayed in Table 1. In total, 117,056 participants, including 54,099 (46.2%) women, were recruited, with the age ranging from 34 to 53 (median, 41) years. According to baseline blood pressure levels, participants were generally divided into normotensive and hypertensive groups. Compared with the normotensive group, the hypertensive group had older age, higher percentages of current smokers and drinkers, and higher levels of BMI, FPG, ALT, AST, BUN, and Scr, whereas percentages of women and family history of diabetes were lower. As for lipid profiles and atherogenic indices, the hypertensive group had higher levels of TC, LDL, non-HDL-c, AC, and AIP, and lower levels of HDL-c, while TG was not significantly different between the two groups.

**Table 1:** Baseline information of the overall population^*^ .

	**Total** **(*n* = 117,056)**	**Normotension** **(*n* = 99,526)**	**Hypertension** **(*n* = 17,530)**	***p* value**
Age (years)	41 (34–53)	39 (33–50)	53 (41–63)	<0.001
Female (%)	54,099 (46.2)	48,304 (48.5)	5,795 (33.1)	<0.001
Current smoker (%)	6,674 (5.7)	5,520 (5.5)	1,154 (6.6)	<0.001
Current drinker (%)	872 (0.7)	651 (0.6)	221 (1.3)	<0.001
Family history of diabetes (%)	2,651 (2.3)	2,367 (2.4)	284 (1.6)	<0.001
BMI (kg/m^2^)	23.2 (21–25.5)	22.9 (20.8–25.1)	25.1 (23–27.3)	<0.001
FPG (mmol/L)	5.0 (4.61–5.35)	4.95 (4.6–5.3)	5.19 (4.8–5.62)	<0.001
ALT (U/L)	18.2 (13–27.8)	18 (12.9–26.8)	22 (15.7–33)	<0.001
AST (U/L)	22.0 (18.7–26.9)	21.8 (18.2–26)	24.2 (20.7–29.7)	<0.001
BUN (mmol/L)	4.57 (3.85–5.4)	4.53 (3.81–5.35)	4.78 (4.06–5.63)	<0.001
Scr (μmol/L)	71.3 (59.3–83.0)	70.5 (58.8–82.4)	75.8 (64.23–86.0)	<0.001
**Lipid profiles and atherosclerotic indices**				
TC (mg/dl)	181.70 (159.67–204.90)	179.77 (158.51–202.97)	191.75 (168.56–216.50)	<0.001
TG (mg/dl)	97.46 (67.34–146.19)	97.46 (67.34–146.19)	97.46 (67.34–147.08)	0.772
LDL (mg/dl)	104.38 (88.53–121.78)	103.22 (87.76–120.62)	110.95 (93.94–128.74)	<0.001
HDL-c (mg/dl)	51.80 (45.23–59.92)	52.19 (45.23–59.92)	51.42 (44.46–59.15)	<0.001
Non-HDL-c (mg/dl)	127.96 (108.25–151.16)	126.03 (106.70–148.84)	138.40 (117.53–162.37)	<0.001
AC	2.43 (1.95–3.1)	2.38 (1.91–3.04)	2.72 (2.21–3.46)	<0.001
AIP	0.28 (0.1–0.47)	0.28 (0.10–0.46)	0.29 (0.11–0.48)	<0.001

* *Continuous data are expressed as median (interquartile range) due to the skewed distribution. The p-value is a comparison between the normotension and hypertension groups. FPG, fasting plasma glucose; TG, triglycerides; TC, total cholesterol; HDL-c, high-density lipoprotein cholesterol; LDL, low-density lipoprotein cholesterol; AC, atherogenic coefficient; AIP, atherogenic risk of plasma; Scr, serum creatinine; BUN, blood urea nitrogen; ALT, alanine aminotransferase; AST, aspartate aminotransferase; BMI, body mass index*.

### Subgroup Analyses of Differences in Lipid Profiles and Atherosclerotic Indices Between Normotensive and Hypertensive Groups

As displayed in Table 2, subgroup analyses stratified by gender, age, BMI, AST, and ALT were conducted. The results of lipid profiles indicated that TC, LDL, and non-HDL-c levels increased significantly in the hypertensive population, regardless of gender, BMI, AST, and ALT levels. Meanwhile, HDL levels decreased significantly in the hypertensive population in some specific subgroups (like women, or BMI <23 kg/m^2^, or AST and ALT <40 U/L). However, in the hypertensive population with BMI ≥ 23 kg/m^2^, their HDL-c levels were mildly higher than those of non-hypertensive people. In all subgroups, there were no significant differences in TC level between the two groups.

**Table 2:** Subgroup analysis of differences in lipid profiles and atherosclerotic indices between normotension and hypertension according to gender, age, BMI, AST, and ALT^*^.

	**Normotension**	**Hypertension**	***p-*value**	**Normotension**	**Hypertension**	***p-*value**
**Gender**	**Male**			**Female**		
TC (mg/dl)	181.70 (160.05–204.90)	189.43 (167.40–212.63)	<0.001	177.84 (158.12–201.80)	199.87 (175.90–225.77)	<0.001
TG (mg/dl)	97.46 (67.34–147.96)	97.46 (66.45–147.08)	0.274	97.46 (68.22–146.19)	99.23 (67.34–147.08)	0.319
LDL (mg/dl)	104.77 (88.92–122.17)	109.02 (92.78–126.80)	<0.001	101.29 (86.21–119.07)	115.59 (98.58–134.54)	<0.001
HDL–c (mg/dl)	49.1 (42.14–56.44)	49.1 (42.14–56.83)	0.073	56.06 (48.71–64.18)	54.51 (47.17–62.63)	<0.001
Non–HDL–c (mg/dl)	131.06 (110.95–154.64)	139.18 (118.30–162.37)	<0.001	120.62 (102.06–143.43)	143.82 (121.59–169.33)	<0.001
AC	2.64 (2.12–3.38)	2.77 (2.25–3.56)	<0.001	2.15 (1.74–2.68)	2.62 (2.14–3.25)	<0.001
AIP	0.31 (0.13–0.49)	0.30 (0.13–0.49)	0.148	0.25 (0.07–0.43)	0.26 (0.09–0.45)	<0.001
**Age**	**<** **60 years**			**≥60 years**		
TC (mg/dl)	177.84 (158.12–201.03)	189.43 (167.01–212.63)	<0.001	197.16 (173.97–222.68)	199.49 (175.90–224.61)	<0.001
TG (mg/dl)	97.46 (67.34–147.08)	97.46 (67.34–145.30)	0.267	97.46 (67.34–147.08)	98.35 (67.34–148.85)	0.349
LDL (mg/dl)	101.68 (86.60–119.07)	109.02 (92.40–126.80)	<0.001	114.82 (97.42–134.15)	115.59 (98.58–134.54)	0.074
HDL-c (mg/dl)	52.19 (45.23–60.70)	50.64 (43.30–58.38)	<0.001	52.58 (44.85–61.08)	51.80 (44.07–60.50)	0.002
Non-HDL-c (mg/dl)	124.10 (105.15–146.91)	137.63 (116.37–160.83)	<0.001	143.82 (121.39–167.40)	146.33 (124.48–170.49)	<0.001
AC	2.34 (1.88–2.99)	2.67 (2.18–3.43)	<0.001	2.72 (2.18–3.42)	2.81 (2.26–3.5)	<0.001
AIP	0.28 (0.1–0.46)	0.29 (0.12–0.48)	<0.001	0.27 (0.1–0.46)	0.28 (0.11–0.47)	0.04
**BMI**	**<** **23 kg/m**^**2**^			**≥23 kg/m** ^**2**^		
TC (mg/dl)	174.36 (154.64–197.17)	189.43 (166.24–214.95)	<0.001	185.57 (164.31–209.54)	193.69 (171.26–218.04)	<0.001
TG (mg/dl)	97.46 (67.34–147.08)	97.46 (66.45–147.96)	0.820	97.46 (67.34–147.08)	97.46 (67.34–146.19)	0.799
LDL (mg/dl)	99.36 (84.67–115.98)	109.02 (92.01–127.19)	<0.001	107.47 (91.62–125.65)	112.11 (95.49–130.28)	<0.001
HDL-c (mg/dl)	55.28 (47.94–63.40)	54.90 (47.17–62.63)	0.007	49.48 (42.53–56.83)	49.87 (42.53–57.60)	<0.001
Non-HDL-c (mg/dl)	117.91 (100.13–139.56)	133.38 (112.11–156.57)	<0.001	135.31 (114.82–158.51)	143.04 (121.78–166.62)	<0.001
AC	2.34 (1.88–2.99)	2.67 (2.18–3.44)	<0.001	2.72 (2.18–3.42)	2.81 (2.27–3.5)	<0.001
AIP	0.28 (0.10–0.46)	0.29 (0.12–0.48)	<0.001	0.27 (0.1–0.46)	0.28 (0.11–0.47)	0.04
**AST**	**<** **40 U/L**			**≥40 U/L**		
TC (mg/dl)	179.0 (158.51–202.19)	190.98 (167.34–216.50)	<0.001	189.43 (166.24–215.72)	197.36 (173.97–227.13)	<0.001
TG (mg/dl)	97.46 (67.34–146.19)	97.46 (67.34–147.08)	0.583	97.46 (67.34–143.98)	98.35 (69.11–150.62)	0.201
LDL (mg/dl)	102.84 (87.76–120.23)	110.57 (93.94–128.35)	<0.001	107.86 (91.24–127.96)	114.63 (95.49–135.31)	<0.001
HDL-c (mg/dl)	52.19 (45.61–60.31)	51.80 (44.85–59.15)	<0.001	50.26 (42.91–57.99)	51.03 (43.30–57.99)	0.510
Non-HDL-c (mg/dl)	125.26 (106.32–148.45)	138.02 (116.75–161.60)	<0.001	138.01 (114.43–162.76)	146.33 (126.03–173.0)	<0.001
AC	2.37 (1.94–2.95)	2.62 (2.19–3.25)	<0.001	2.68 (2.16–3.48)	2.84 (2.39–3.61)	<0.001
AIP	0.27 (0.10–0.46)	0.28 (0.11–0.47)	0.004	0.29 (0.12–0.48)	0.31 (0.13–0.50)	0.263
**ALT**	**<** **40 U/L**			**≥40 U/L**		
TC (mg/dl)	178.22 (158.51–201.42)	192.14 (169.33–216.50)	<0.001	192.14 (169.72–216.50)	197.55 (175.90–222.68)	<0.001
TG (mg/dl)	178.22 (158.51–201.12)	192.14 (169.33–216.50)	0.547	97.46 (67.34–145.30)	97.46 (69.11–143.53)	0.540
LDL (mg/dl)	102.06 (86.99–119.46)	110.57 (94.33–128.74)	<0.001	110.58 (93.94–129.51)	114.43 (96.65–132.99)	<0.001
HDL–c (mg/dl)	52.58 (45.62–61.08)	51.42 (44.07–59.54)	<0.001	47.94 (40.98–55.67)	48.32 (40.98–56.06)	0.411
Non–HDL–c (mg/dl)	124.49 (105.16–147.29)	139.18 (117.91–162.76)	<0.001	142.66 (120.62–167.40)	148.45 (127.0–172.42)	<0.001
AC	2.33 (1.87–2.95)	2.67 (2.17–3.37)	<0.001	2.92 (2.32–3.80)	3.0 (2.43–3.93)	<0.001
AIP	0.27 (0.1–0.46)	0.28 (0.11–0.47)	<0.001	0.31 (0.14–0.50)	0.31 (0.14–0.5)	0.764

* *Continuous data are expressed as median (interquartile range) due to the skewed distribution. TG, triglycerides; TC, total cholesterol; HDL–c, high–density lipoprotein cholesterol; LDL, low–density lipoprotein cholesterol; AC, atherogenic coefficient; AIP, atherogenic risk of plasma; ALT, alanine aminotransferase; AST, aspartate aminotransferase; BMI, body mass index*.

Comparisons of the atherosclerotic indices in the normotensive and hypertensive groups indicated that AC was significantly increased in the hypertensive population, regardless of gender and gender, age, BMI, AST, and ALT levels. Similarly, AIP was higher in the hypertensive population regardless of age and BMI yet this association was not observed in men and in those with elevated AST and ALT.

### Multivariate Logistic Analyses of Lipid Profiles, and Atherosclerotic Indices and the Incidence of Hypertension

As shown in Table 3, TC, TG, LDL, HDL-c, non-HDL-c, AC, and AIP were treated as the continuous variables and divided into quartiles, with the first quartile as a reference group. In the crude model, no covariables were adjusted; in model 1, age and gender were adjusted; in model 2, all the covariables, including BMI, FPG, ALT, AST, BUN, Scr, smoking status, drinking status, and family history of diabetes, were fully adjusted. In the crude model, there was no significant increase in the risk of hypertension with increasing TG level (*p* for trend = 0.767), and the results were maintained after adjusting for full covariables (*p* for trend = 0.844). However, in the crude model, the risk of hypertension increased significantly as the levels of TC, LDL, HDL-c, non-HDL-c, AC, and AIP increased (*p* for trend <0.001), and this association persisted after adjusting for gender and age, except for AIP.

**Table 3:** Multivariable logistic regression model evaluating the association between serum lipid levels and hypertension.

	**Crude model**		**Model 1**		**Model 2**	
	**OR (95% CI)**	***p-*value**	**OR (95% CI)**	***p-*value**	**OR (95% CI)**	***p-*value**
**TG**						
Q 1 ( ≤ 67.3 mg/dl)	Ref					
Q 2 (67.3–97.5 mg/dl)	0.967 (0.923–1.012)	0.149	0.979 (0.932–1.028)	0.394	0.960 (0.889–1.037)	0.296
Q 3 (97.5–147.1 mg/dl)	0.995 (0.951–1.040)	0.822	0.993 (0.947–1.042)	0.781	1.007 (0.934–1.085)	0.862
Q 4 (≥147.1 mg/dl)	0.984 (0.940–1.029)	0.478	0.979 (0.933–1.027)	0.378	0.993 (0.920–1.071)	0.849
Per 1 mg/dl increase	1.000 (1.000–1.000)	0.624	1.000 (1.000–1.000)	0.938	1.000 (1.000–1.000)	0.160
*p* for trend	0.767		0.512		0.844	
**TC**						
Q 1 ( ≤ 160.4 mg/dl)	Ref					
Q 2 (160.4–181.7 mg/dl)	1.355 (1.286–1.428)	<0.001	1.178 (1.115–1.245)	<0.001	0.979 (0.901–1.065)	0.31
Q 3 (181.7–205.7 mg/dl)	1.821 (1.733–1.913)	<0.001	1.382 (1.312–1.457)	<0.001	1.093 (1.008–1.184)	0.013
Q 4 (≥205.7 mg/dl)	2.529 (2.411–2.653)	<0.001	1.561 (1.483–1.643)	<0.001	1.105 (1.021–1.196)	<0.001
Per 1 mg/dl increase	1.010 (1.009–1.010)	<0.001	1.005 (1.004–1.006)	<0.001	1.002 (1.001–1.003)	<0.001
*p* for trend	<0.001		<0.001		<0.001	
**LDL**						
Q 1 ( ≤ 88.5 mg/dl)	Ref					
Q 2 (67.3–104.4 mg/dl)	1.322 (1.257–1.391)	<0.001	1.143 (1.083–1.206)	<0.001	0.979 (0.901–1.065)	0.625
Q 3 (104.4–122.2 mg/dl)	1.671 (1.592–1.755)	<0.001	1.283 (1.218–1.351)	<0.001	1.093 (1.008–1.184)	0.031
Q 4 (≥122.2 mg/dl)	2.208 (2.107–2.314)	<0.001	1.402 (1.334–1.475)	<0.001	1.105 (1.021–1.196)	0.013
Per 1 mg/dl increase	1.010 (1.009–1.011)		1.005 (1.004–1.006)		1.002 (1.001–1.003)	<0.001
*p* for trend	<0.001		<0.001		0.001	
**HDL-c**						
Q 1 ( ≤ 44.8 mg/dl)	Ref					
Q 2 (44.8–52.2 mg/dl)	0.838 (0.801–0.875)	<0.001	0.948 (0.904–0.994)	0.026	1.091 (1.012–1.176)	0.023
Q 3 (52.2–60.3 mg/dl)	0.801 (0.7666–0.837)	<0.001	0.947 (0.903–0.994)	0.026	1.194 (1.107–1.288)	<0.001
Q 4 (≥60.3 mg/dl)	0.719 (0.687–0.752)	<0.001	0.877 (0.835–0.922)	<0.001	1.192 (1.100–1.292)	<0.001
Per 1 mg/dl increase	0.989 (0.988–0.990)	<0.001	0.995 (0.994–0.997)	<0.001	1.006 (1.003–1.008)	<0.001
*p* for trend	<0.001		<0.001		<0.001	
**Non-HDL-c**						
Q 1 ( ≤ 107.9 mg/dl)	Ref					
Q 2 (107.9–128 mg/dl)	1.558 (1.476–1.664)	<0.001	1.267 (1.197–1.341)	<0.001	1.042 (0.955–1.137)	0.354
Q 3 (128–152.3 mg/dl)	2.186 (2.077–2.301)	<0.001	1.485 (1.407–1.568)	<0.001	1.144 (1.052–1.244)	0.002
Q 4 (≥152.3 mg/dl)	3.059 (2.911–3.214)	<0.001	1.708 (1.620–1.8)	<0.001	1.148 (1.056–1.248)	0.001
Per 1 mg/dl increase	1.011 (1.010–1.012)	<0.001	1.006 (1.005–1.007)	<0.001	1.002 (1.001–1.003)	<0.001
*p* for trend	<0.001		<0.001		<0.001	
**AC**						
Q 1 ( ≤ 1.95)	Ref		Ref		Ref	
Q 2 (1.95–2.43)	1.756 (1.664–1.853)	<0.001	1.428 (1.350–1.511)	<0.001	1.193 (1.091–1.305)	<0.001
Q 3 (2.43–3.10)	2.373 (2.253–2.498)	<0.001	1.595 (1.510–1.685)	<0.001	1.205 (1.104–1.315)	<0.001
Q 4 (≥3.10)	2.962 (2.816–3.115)	<0.001	1.712 (1.621–1.807)	<0.001	1.084 (0.99–1.187)	0.083
Per 1-unit increase	1.350 (1.331–1.370)	<0.001	1.148 (1.130–1.167)	<0.001	0.981 (0.954–1.009)	0.178
*p* for trend	<0.001		<0.001		0.459	
**AIP**						
Q 1 ( ≤ 0.104)	Ref		Ref		Ref	
Q 2 (0.104–0.279)	1.073 (1.025–1.124)	0.003	1.038 (0.989–1.091)	0.131	1.012 (0.937–1.093)	0.757
Q 3 (0.279–0.466)	1.099 (1.050–1.151)	<0.001	1.037 (0.988–1.090)	0.141	0.990 (0.917–1.069)	0.805
Q 4 (≥0.466)	1.128 (1.077–1.180)	<0.001	1.042 (0.992–1.094)	0.10	0.971 (0.899–1.049)	0.453
Per 0.1-unit increase	1.179 (1.111–1.251)	<0.001	1.044 (0.980–1.113)	0.184	0.960 (0.868–1.063)	0.437
*p* for trend	<0.001		0.127		0.367	

*OR, odds ratio; CI, confidence interval; Q, quartile. Model 1 adjust age and gender. Model 2 adjust Model 1+ BMI (kg/m^2^), FPG (mmol/L), ALT (U/L), AST (U/L), BUN, Scr, smoking status (current smoker or not), drinking status (current drinker or not), family history of diabetes (Yes or No)*.

In model 2, after fully adjusting for all the covariates, the incidence of hypertension increased by 0.2% (1.002[1.001–1.003]), 0.2% (1.002[1.001–1.003]), and 0.2% (1.002[1.001–1.003]), respectively, per 1 mg/dl increase in TC, LDL, and non-HDL. Surprisingly, after fully adjusting for covariates, an increase in HDL was associated with a higher risk of hypertension (*p* for trend <0.001), to be specific, the risk of hypertension increased by 0.6% (1.006[1.003–1.008]) with a 1 mg/dl increase in HDL-c. In addition, after fully adjustment for all variables, AIP was not significantly associated with the incidence of hypertension. AC was positively associated with the incidence of hypertension only at less than 3.1, and the trend p for AC and risk of hypertension was 0.459.

### Predictive Value of AC and AIP for the Incidence of Hypertension

As shown in Table 4, AC had a predictive value for the incidence of hypertension, regardless of age, sex, BMI, AST, and ALT. In women, AC had the highest predictive value for the incidence of hypertension with an area under the curve (AUC) of 0.667 [95% confidence interval (CI): 0.659–0.674]. However, the predictive value of AIP for the incidence of hypertension was low.

**Table 4:** Predictive value of AC and AIP for the incidence of hypertension according to BMI, age, gender, AST and ALT.

		**AUC**	**95%CI**	***p*–value**
**BMI** **<** **23 kg/m**^**2**^	AC	0.607	0.598–0.616	<0.001
	AIP	0.503	0.494–0.512	0.550
**BMI** **≥23 kg/m**^**2**^	AC	0.541	0.536–0.547	<0.001
	AIP	0.496	0.491–0.502	0.181
**Age** **<** **60 years**	AC	0.612	0.607–0.618	<0.001
	AIP	0.515	0.510–0.521	<0.001
**Age** **≥60 years**	AC	0.527	0.518–0.536	<0.001
	AIP	0.509	0.500–0.518	0.04
**Male**	AC	0.545	0.540–0.551	<0.001
	AIP	0.496	0.490–0.502	0.148
**Female**	AC	0.667	0.659–0.674	<0.001
	AIP	0.517	0.509–0.525	<0.001
**AST** **<** **40U/L**	AC	0.596	0.589–0.603	<0.001
	AIP	0.511	0.504–0.518	0.004
**AST** **≥40U/L**	AC	0.558	0.531–0.584	<0.001
	AIP	0.516	0.488–0.544	0.263
**ALT** **<** **40U/L**	AC	0.613	0.608–0.618	<0.001
	AIP	0.511	0.506–0.516	<0.001
**ALT** **≥40U/L**	AC	0.530	0.519–0.542	<0.001
	AIP	0.502	0.490–0.514	0.763

*AUC, area under curve; CI, confidence interval; AC, atherogenic coefficient; AIP, atherogenic risk of plasma; ALT, alanine aminotransferase; AST, aspartate aminotransferase; BMI, body mass index*.

### Differences in Lipid Profiles, and Atherosclerotic Indices Between Normotensive and Hypertensive Groups

**Figure 2:**
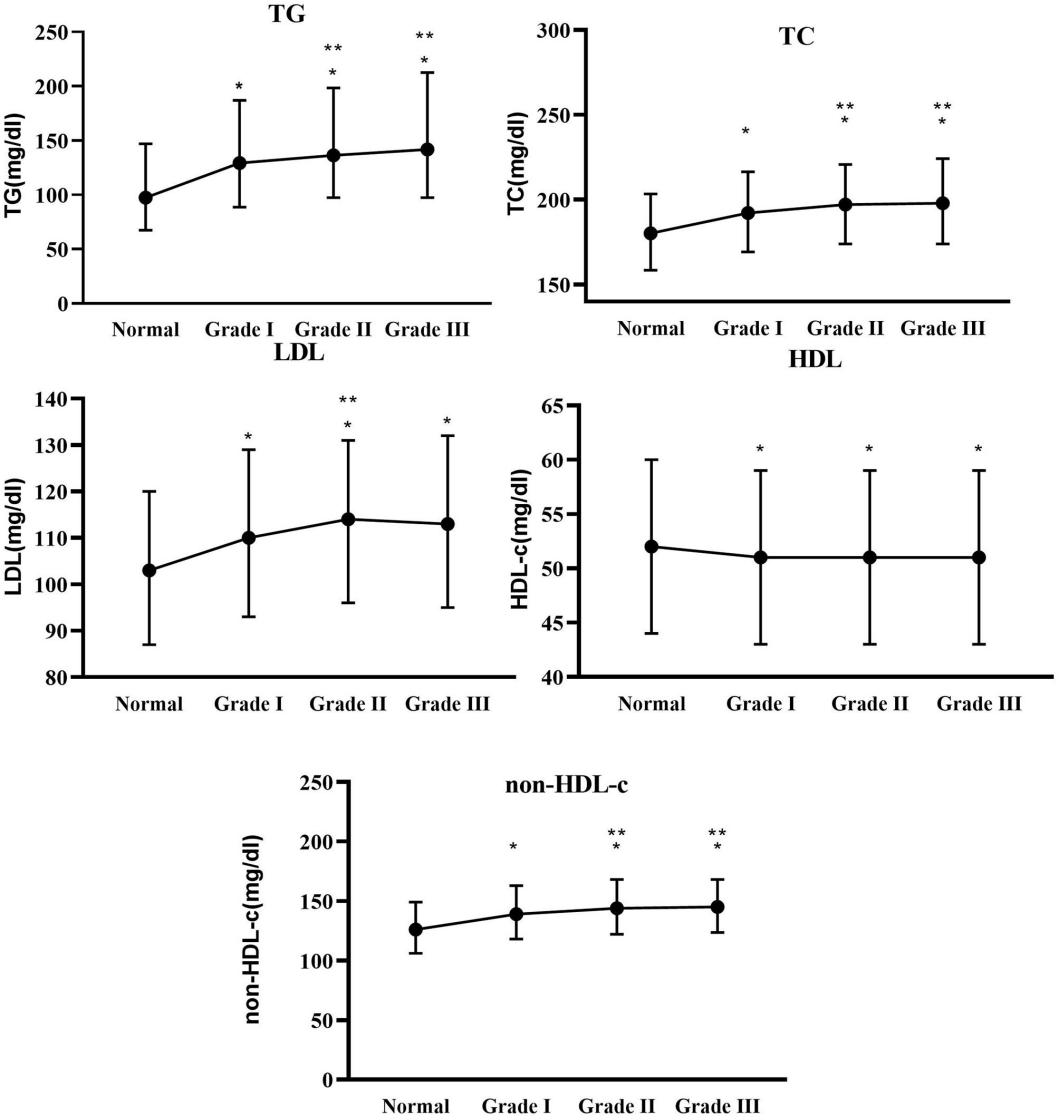
Between-group differences in total cholesterol (TC), TG, low-density lipoprotein (LDL), high-density lipoprotein cholesterol (HDL-c), and non-HDL-c at different blood pressure levels. * Indicates Grades I–III compared with the normal group with *p* < 0.05. ** Indicates Grades II and III compared with Grade I with *p* < 0.05.

Based on the baseline blood pressure levels, hypertension was further classified into Grades I–III. As shown in Figure 2, TG, TC, and non-HDL-c levels were significantly increased in Grades I–III groups compared with the normotensive group (*p* < 0.05), whereas TG, TC, and non-HDL-c levels significantly increased in Grades II and III groups relative to the Grade I group (*p* < 0.05). Similarly, HDL-c and LDL levels were significantly higher in Grades I–III groups than in the normotensive group (*p* < 0.05), while LDL levels were higher in the Grade II group than in the Grade I group (*p* < 0.05).

**Figure 3:**
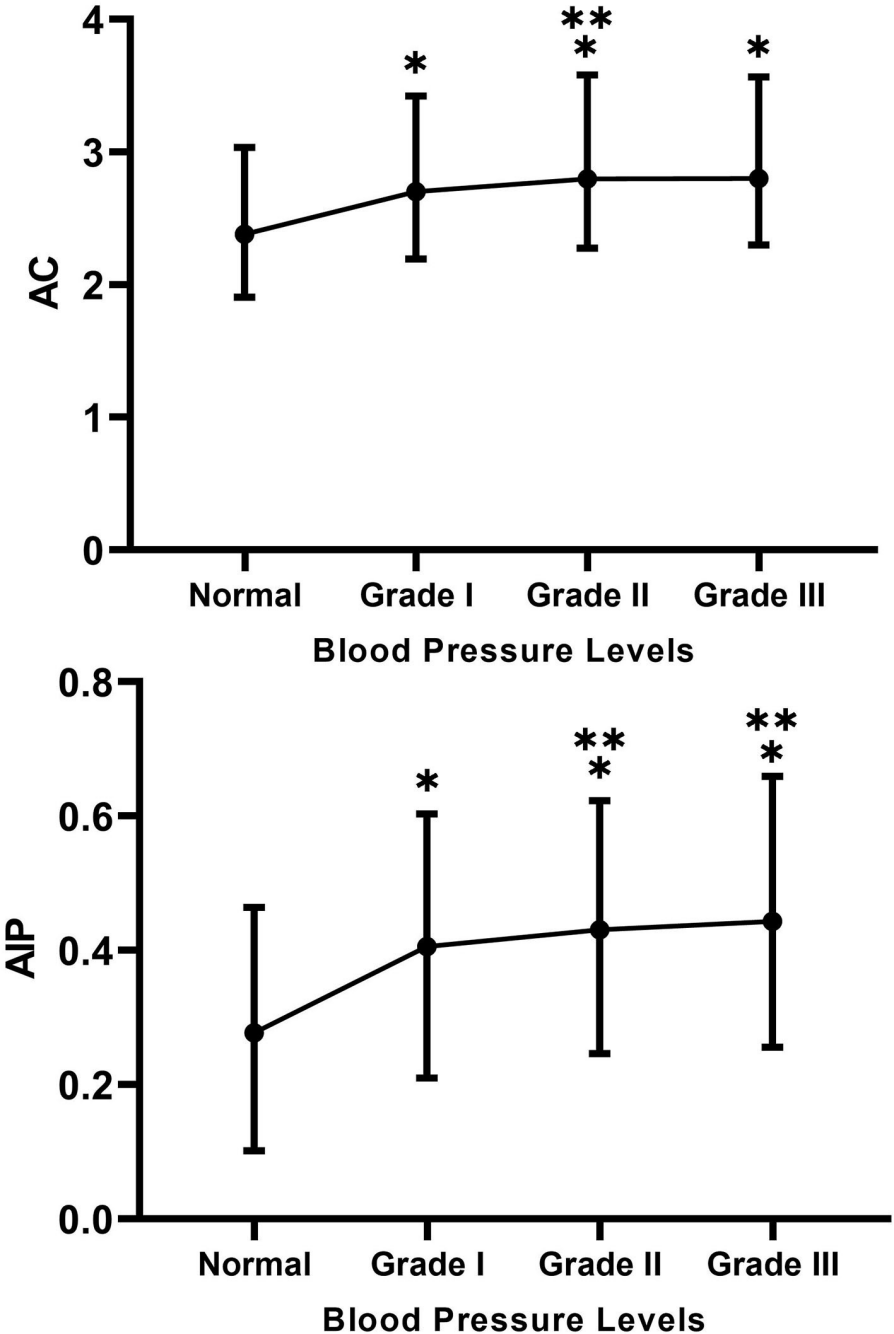
Between-group differences in atherogenic coefficient (AC) and atherogenic risk of plasma (AIP) at different blood pressure levels. * Indicates Grades I–III compared with the normal group with *p* < 0.05. ** Indicates Grades II and III compared with Grade I with *p* < 0.05.

Similarly, as shown in Figure 3, AC and AIP were significantly higher in Grades I–III groups compared with the normotensive group (*p* < 0.05), while AC and AIP were higher in the Grade II group compared with the Grade I group (*p* < 0.05). However, only a higher AIP was observed in the Grade III group than in the Grade I group (*p* < 0.05).

### BN Analysis of the Relationship Between Baseline Characteristics, Lipid Profiles, and Atherosclerotic Indices and the Incidence of Hypertension

In the BN model, each circle represents a predictor, and the depth of its color indicates the importance for the occurrence of hypertension, with a darker color indicating higher importance. When two predictors are connected along the arrows, it indicates the existence of a direct relationship; otherwise, it indicates an indirect relationship.

**Figure 4:**
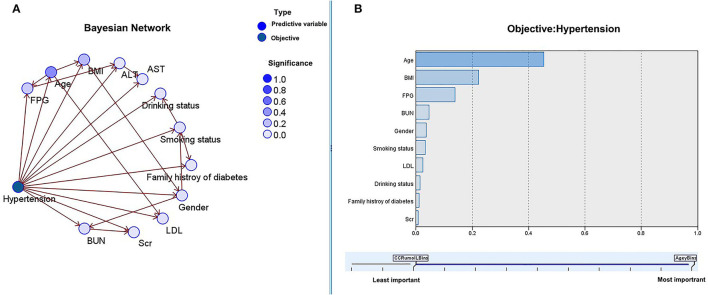
The Bayesian model based on the TAN algorithm: the relationship between baseline characteristics and the incidence of hypertension **(A)** and the importance of predictors **(B)**.

**Figure 5:**
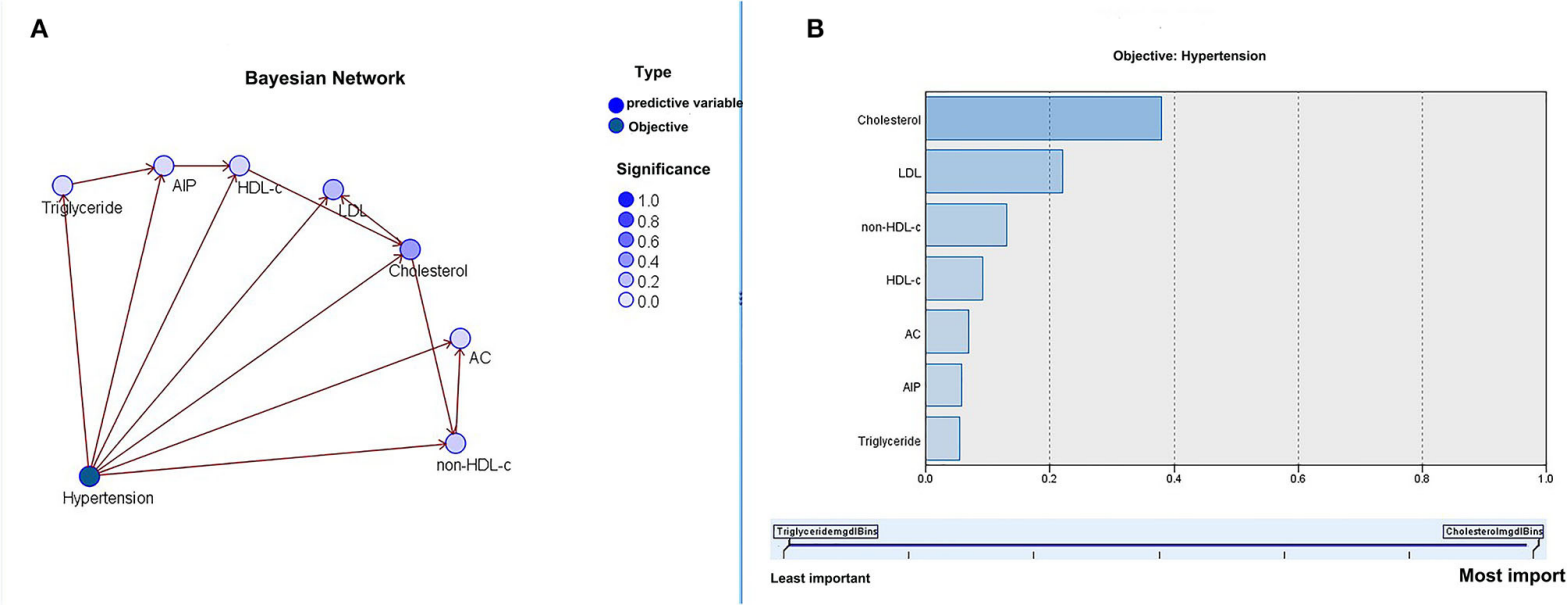
The Bayesian model based on the tree augmented native (TAN) algorithm: the relationship between lipid profiles, atherosclerotic indices, and the incidence of hypertension **(A)** and importance of predictors **(B)**.

As shown in Figures 4A,B, the results of the BN model showed that, among the baseline characteristics of the participants, age, BMI, and FPG indirectly affected the incidence of hypertension, whereas age directly affected BMI and FPG. Age, BMI, and FPG had a greater effect on the incidence of hypertension. In addition, as shown in Figures 5A,B, in terms of lipids and atherosclerotic indices, TC and LDL levels were indirectly related to the incidence of hypertension, while TC directly influenced LDL levels. TC and LDL were more closely related to the incidence of hypertension.

## Discussion

This cross-sectional study was conducted based on data collected from 32 healthcare centers in 11 Chinese cities and included a total of 117,056 participants. The following results were obtained in this study. (1) There were significant differences in baseline information and lipid profiles between normotensive and hypertensive groups. (2) Lipid profiles were higher in the hypertensive population, with higher serum levels of TC, LDL, non-HDL-c, and lower HDL-c levels. AC and AIP were higher in the hypertensive population. (3) Subgroup analyses also reached similar results, but HDL-c levels were increased in the hypertensive population with BMI ≥ 23 kg/m^2^. (4) In addition, in the hypertensive population, AIP was not significantly increased in men and in those with elevated AST and ALT.

In multivariable logistic regression, HDL-c levels were positively associated with the risk of hypertension after adjusting for BMI; AIP was not associated with the incidence of hypertension; AC was positively associated with the incidence of hypertension only at less than 3.1. (5) TG, TC, LDL, non-HDL-c, AC, and AIP were higher in the hypertensive population (regardless of the grade) than in the normotensive group, while HDL-c levels were lower than in the normotensive group. (6) The results of the BN model showed that age, BMI, and FPG were strongly associated with the incidence of hypertension among baseline characteristics. The BN model of lipid profile and atherosclerotic indices showed that TC and LDL were closely associated with the risk of hypertension. (7) AP has a good predictive value for the incidence of hypertension, with the highest predictive value in women.

Dyslipidemia is strongly associated with the development of hypertension. Previous cross-sectional studies have supported the hypothesis of a biological interrelationship between blood pressure and lipids (18, 19), while the relationship between lipid profiles and the risk of new-onset hypertension is further confirmed in cohort studies (20, 21). Nevertheless, different patterns of dyslipidemia have exhibited inconsistent results across diverse ethnic groups. As reported in an East Asian cohort study, HDL-c exerts a protective effect on the risk of hypertension in women, whereas TC and TG were not significantly associated with the risk of hypertension (22). Additionally, a cohort study based on 16,130 US women shows a protective effect of HDL-c on the risk of hypertension, a positive association of non-HDL-c with the risk of hypertension, and no significant association between LDL and the risk of hypertension (9). Also, a study based on 14,215 Japanese men suggests that elevated serum TC, LDL, and non-HDL-c levels are associated with an increased risk of hypertension, whereas there is no significant evidence to support the relation between elevated TG levels and the risk of hypertension. Meanwhile, Otsuka et al. suggested that HDL-c levels were not linearly related to the risk of hypertension, but rather in a U-shaped curve (11). To some extent, the above findings are similar to the results of our study (11). Recently, a study from China shows that HDL-c is negatively associated with the risk of hypertension in women, but not in men. Meanwhile, the protective effect of HDL-c on the risk of hypertension is only found in people less than 60 years, and HDL-c shows a positive association with the risk of hypertension after adjusting for BMI, consistent with our findings (23). At the same time, another study from China discovers that low or high HDL-c levels are associated with a high risk of cardiovascular events (24). In China, compared with normotensive individuals, patients with hypertension over 60 years may have other CVDs, and those with a BMI > 23 kg/m^2^ tend to suffer from some metabolic diseases. These individuals may have undergone early lifestyle and pharmacological interventions, such as low-salt and low-fat diet, antihypertensive therapy, lipid-lowering therapy, and improvement of endothelial function, which may passively increase HDL-c levels. Indeed, the anti-atherosclerotic function of HDL-c is achieved by means of reverse cholesterol transport, maintenance of endothelial cell homeostasis, and its potent antioxidant properties (25, 26). Moreover, randomized clinical trials (RCTs) have confirmed that there is no additional benefit from the pharmacological increase of HDL-c (27). This may partly explain the elevated HDL-c levels in the specific hypertensive population.

Atherogenic coefficient and AIP, as predictors of arteriosclerosis, have been widely used in CVD screening and risk stratification (14, 28–30), while relevant studies in the hypertensive population are still limited (31, 32). In this study, we observed that AC and AIP were higher in the hypertensive population than in the normotensive population, which was associated with an increased proportion of dyslipidemia in the hypertensive population. However, in multivariate logistic regression, AIP was not significantly associated with the incidence of hypertension, which is consistent with the findings of Choudhary et al. (33). In addition, in the analysis of AC and AIP to predict the incidence of hypertension, we found a good predictive value of AC, especially in women. In Table 2, we can find that TC was significantly higher in women than in the other groups, which may partly explain the better prediction of the incidence of hypertension by AC in women. The mechanism behind this remains unclear, and we speculate that a significant increase in TC may be related to female hormone levels.

In addition, from the results of this study, we found no obvious evidence to support the association of TG with an increased risk of hypertension, which was inconsistent with previous studies (34, 35). It is well known that TG reflects the degree of visceral fat accumulation (36). However, visceral adipose tissues should differ between countries due to differences in race, diet, and lifestyle (1). Nazare et al. reported that the visceral adipose tissues of the Chinese differed significantly from those of European and US populations, so there might be differences in the effect of TG on the risk of hypertension (37). In the BN model analysis, TC had the highest importance value on the effect of hypertension, and TC directly affected LDL; as a result, TC might play a “pivotal” role in the development of lipids and hypertension. Indeed, in the Framingham Study, Castelli et al. and Anderson et al. found a strong correlation between blood pressure and serum cholesterol in patients with hypertension. They also suggested the need for early treatment of hypercholesterolemia in these patients with hypertension (38, 39). As well, Gaziano et al. pointed out a potential interaction, rather than independence, between TC and hypertension in the development of myocardial infarction (MI) (40).

Lipids may be involved in the following biological mechanisms in the pathogenesis of hypertension. (1) Lipid abnormalities may act on vascular endothelial cells and affect the production, release, and function of nitric oxide (NO), which may lead to atherosclerosis (41). (2) Dyslipidemia can lead to smooth muscle cell hypertrophy and collagen deposition, which further aggravates arterial wall atherosclerosis. Concurrently, dyslipidemia can induce renal capillary damage, resulting in the development of secondary hypertension (42). (3) There are some genes that identically interact between hyperlipidemia and hypertension, such as apolipoprotein (apo) A-I, apo e, microsomal TG transporter protein, and lipoprotein lipase genes (43). (4) Lipid abnormalities and insulin resistance are associated with sympathetic hyperfunction, and the latter may be related to the development of hypertension (44).

The following limitations should be noted in this work. (1) First, this is a cross-sectional study and our results do not support a causal relationship. (2) Second, the population studied was mainly from eastern China, and the results in this paper were only partially representative of this region. (3) This study did not collect details on medication use or other chronic disease conditions that might partially affect the results of lipids and the risk of hypertension, this deficiency should be improved in the design of future studies.

## Conclusions

Overall, compared with the normotensive group, TC, TG, LDL-c, and non-HDL-c levels are significantly higher, while HDL-c levels are significantly lower in the hypertensive group. TC and LDL were strongly associated with the incidence of hypertension. TC, LDL, and non-HDL-c levels show a positive association, HDL-c shows a negative association, while TG is not significantly associated with the risk of hypertension. After adjusting for BMI, HDL-c turns out to be positively associated with the risk of hypertension. In addition, AC has a good predictive value for the incidence of hypertension in women.

## Data Availability Statement

The datasets presented in this study can be found in online repositories. The names of the repository/repositories and accession number(s) can be found below: https://datadryad.org/stash/dataset/doi:10.5061/dryad.ft8750v.

## Ethics Statement

The studies involving human participants were reviewed and approved by Rich Healthcare Group Review Board. Written informed consent for participation was not required for this study in accordance with the national legislation and the institutional requirements.

## Author Contributions

WKC and SWC designed. WKC drafted, analyzed, and interpreted this study. WKC, LLW, and SWC critically reviewed the study. All authors finally agreed and read and approved the submitted manuscript.

## Funding

WKC is funded by the China Scholarship Council (CSC No. 202009370095).

## Conflict of Interest

The authors declare that the research was conducted in the absence of any commercial or financial relationships that could be construed as a potential conflict of interest.

## Publisher's Note

All claims expressed in this article are solely those of the authors and do not necessarily represent those of their affiliated organizations, or those of the publisher, the editors and the reviewers. Any product that may be evaluated in this article, or claim that may be made by its manufacturer, is not guaranteed or endorsed by the publisher.

## References

[1] Huang J Bao X Xie Y Zhang X Peng X Liu Y. Interaction of lipid accumulation product and family history of hypertension on hypertension risk: a cross-sectional study in the Southern Chinese population. BMJ Open. (2019) 9:e029253. 10.1136/bmjopen-2019-029253 31784431 PMC6924775

[2] Kaneva AM Bojko ER. Sex differences in the association between obesity and hypertension. Arch Physiol Biochem. (2021) 21:1–8. 10.1080/13813455.2020.1861027 33476209

[3] Kearney PM Whelton M Reynolds K Muntner P Whelton PK He J. Global burden of hypertension: analysis of worldwide data. Lancet. (2005) 365:217–23. 10.1016/S0140-6736(05)17741-1 15652604

[4] Beaney T Schutte AE Tomaszewski M Ariti C Burrell LM Castillo RR. May Measurement Month 2017: an analysis of blood pressure screening results worldwide. Lancet Glob Health. (2018) 6:e736–e743. 10.1016/s2214-109x(18)30259-6 29778399

[5] Bundy JD He J. Hypertension and related cardiovascular disease burden in China. Ann Glob Health. (2016) 82:227–33. 10.1016/j.aogh.2016.02.002 27372527

[6] He D Fan F Jia J Jiang Y Sun P Wu Z. Lipid profiles and the risk of new-onset hypertension in a Chinese community-based cohort. Nutr Metab Cardiovasc Dis. (2021) 31:911–20. 10.1016/j.numecd.2020.11.026 33549431

[7] Varughese GI Patel JV Lip GY Varma C. Novel concepts of statin therapy for cardiovascular risk reduction in hypertension. Curr Pharm Des. (2006) 12:1593–609. 10.2174/138161206776843304 16729872

[8] Neaton JD Wentworth D. Serum cholesterol, blood pressure, cigarette smoking, and death from coronary heart disease. Overall findings and differences by age for 316,099 white men multiple risk factor intervention trial research group. Arch Intern Med. (1992) 152:56–64. 10.1001/archinte.152.1.56 1728930

[9] Sesso HD Buring JE Chown MJ Ridker PM Gaziano JM. A prospective study of plasma lipid levels and hypertension in women. Arch Intern Med. (2005) 165:2420–7. 10.1001/archinte.165.20.2420 16287773

[10] Laaksonen DE Niskanen L Nyyssönen K Lakka TA Laukkanen JA Salonen JT. Dyslipidaemia as a predictor of hypertension in middle-aged men. Eur Heart J. (2008) 29:2561–8. 10.1093/eurheartj/ehn061 18308688 PMC2721716

[11] Otsuka T Takada H Nishiyama Y Kodani E Saiki Y Kato K. Dyslipidemia and the risk of developing hypertension in a working-age male population. J Am Heart Assoc. (2016) 5:e003053. 10.1161/JAHA.115.003053 27016576 PMC4943276

[12] Chen Y Zhang X-P Yuan J Cai B Wang X-L Wu X-L. Association of body mass index and age with incident diabetes in Chinese adults: a population-based cohort study. BMJ Open. (2018) 8:e021768. 10.1136/bmjopen-2018-021768 30269064 PMC6169758

[13] Wu Y Hu H Cai J Chen R Zuo X Cheng H. Association of hypertension and incident diabetes in Chinese adults: a retrospective cohort study using propensity-score matching. BMC Endocr Disord. (2021) 21:87. 10.1186/s12902-021-00747-0 33926442 PMC8082672

[14] Sujatha R Kavitha S. Atherogenic indices in stroke patients: a retrospective study. Iran J Neurol. (2017) 16:78–82. 28761629 PMC5526781

[15] Guidelines for the Prevention and Treatment of Hypertension in China (2018 Revised Edition). Chin J Cardiovasc Med. (2019) 24:24–56.

[16] Guo S He J Li J Tang B. Exploring the impact of unsafe behaviors on building construction accidents using a bayesian network. Int J Environ Res Public Health. (2019) 17. 10.3390/ijerph17010221 31892270 PMC6981992

[17] Chien P-L Liu C-F Huang H-T Jou H-J Chen S-M Young T-G. Application of artificial intelligence in the establishment of an association model between metabolic syndrome, TCM constitution, and the guidance of medicated diet care. Evid Based Complement Alternat Med. (2021) 2021:5530717. 10.1155/2021/5530717 34007288 PMC8110390

[18] Choudhury KN Mainuddin AK Wahiduzzaman M Islam SM. Serum lipid profile and its association with hypertension in Bangladesh. Vasc Health Risk Manag. (2014) 10:327–32. 10.2147/VHRM.S61019 25061312 PMC4086853

[19] Akintunde AA Ayodele EO Akinwusi OP Opadijo GO. Dyslipidemia among newly diagnosed hypertensives: pattern and clinical correlates. J Natl Med Assoc. (2010) 102:403–7. 10.1016/S0027-9684(15)30575-7 20533775

[20] Halperin RO Sesso HD Ma J Buring JE Stampfer MJ Gaziano JM. Dyslipidemia and the risk of incident hypertension in men. Hypertension. (2006) 47:45–50. 10.1161/01.HYP.0000196306.42418.0e 16344375

[21] Sánchez-Íñigo L Navarro-González D Pastrana-Delgado J Fernández-Montero A Martínez JA. Association of triglycerides and new lipid markers with the incidence of hypertension in a Spanish cohort. J Hypertens. (2016) 34:1257–65. 10.1097/HJH.0000000000000941 27136314

[22] Bozorgmanesh M Ghoreishian H Mohebi R Azizi F Hadaegh F. Sex-specific predictors of the prehypertension-to-hypertension progression: community-based cohort of a West-Asian population. Eur J Prev Cardiol. (2014) 21:956–63. 10.1177/2047487313481757 23478742

[23] Yang G Qian T Sun H Xu Q Hou X Hu W. Adjustment for body mass index changes inverse associations of HDL-cholesterol with blood pressure and hypertension to positive associations. J Hum Hypertens. (2021). 10.1038/s41371-021-00548-x 33976343

[24] Yu S Guo X Li GX Yang H Zheng L Sun Y. Lower or higher HDL-c levels are associated with cardiovascular events in the general population in rural China. Lipids Health Dis. (2020) 19:152. 10.1186/s12944-020-01331-6 32586331 PMC7315555

[25] Mineo C Deguchi H Griffin JH Shaul PW. Endothelial and antithrombotic actions of HDL. Circ Res. (2006) 98:1352–64. 10.1161/01.RES.0000225982.01988.93 16763172

[26] Barter PJ Nicholls S Rye K-A Anantharamaiah GM Navab M Fogelman AM. Antiinflammatory properties of HDL. Circ Res. (2004) 95:764–72. 10.1161/01.RES.0000146094.59640.13 15486323

[27] Keene D Price C Shun-Shin MJ Francis DP. Effect on cardiovascular risk of high density lipoprotein targeted drug treatments niacin, fibrates, and CETP inhibitors: meta-analysis of randomised controlled trials including 117,411 patients. BMJ. (2014) 349:g4379. 10.1136/bmj.g4379 25038074 PMC4103514

[28] Dobiásová M. Atherogenic index of plasma log(triglycerides/HDL-cholesterol): theoretical and practical implications. Clin Chem. (2004) 50:1113–5. 10.1373/clinchem.2004.033175 15229146

[29] Olamoyegun MA Oluyombo R Asaolu SO. Evaluation of dyslipidemia, lipid ratios, and atherogenic index as cardiovascular risk factors among semi-urban dwellers in Nigeria. Ann Afr Med. (2016) 15:194–9. 10.4103/1596-3519.194280 27853034 PMC5402827

[30] Frohlich J Dobiásová M. Fractional esterification rate of cholesterol and ratio of triglycerides to HDL-cholesterol are powerful predictors of positive findings on coronary angiography. Clin Chem. (2003) 49:1873–80. 10.1373/clinchem.2003.022558 14578319

[31] Yin J Li M Yu L Hu F Yu Y Hu L. The relationship between the atherogenic index of plasma and arterial stiffness in essential hypertensive patients from China: a cross-sectional study. BMC Cardiovasc Disord. (2021) 21:245. 10.1186/s12872-021-02049-8 34011265 PMC8136204

[32] Rodríguez-Morán M Guerrero-Romero F Aradillas-García C Bermudez-Peña C Simental-Mendia LE Vargas Morales JM. Atherogenic indices and prehypertension in obese and non-obese children. Diab Vasc Dis Res. (2013) 10:17–24. 10.1177/1479164112440713 22441379

[33] Choudhary MK Eräranta A Koskela J Tikkakoski AJ Nevalainen PI Kähönen M. Atherogenic index of plasma is related to arterial stiffness but not to blood pressure in normotensive and never-treated hypertensive subjects. Blood Press. (2019) 28:157–67. 10.1080/08037051.2019.1583060 30821503

[34] Osuji CU Omejua EG Onwubuya EI Ahaneku GI. Serum lipid profile of newly diagnosed hypertensive patients in nnewi, South-East Nigeria. Int J Hypertens. (2012) 2012:710486. 10.1155/2012/710486 23304451 PMC3523141

[35] Hadaegh A Akbarpour S Tohidi M Barzegar N Hosseinpour-Niazi S Azizi F. The role of different lipid measures for incident hypertension during more than 12-year follow-up: Tehran Lipid and Glucose Study. Br J Nutr. (2021) 23:1–11. 10.1017/S0007114521004657 34809728

[36] Nurdiantami Y Watanabe K Tanaka E Pradono J Anme T. Association of general and central obesity with hypertension. Clin Nutr. (2018) 37:1259–63. 10.1016/j.clnu.2017.05.012 28583324

[37] Nazare J-A Smith JD Borel A-L Haffner SM Balkau B Ross R. Ethnic influences on the relations between abdominal subcutaneous and visceral adiposity, liver fat, and cardiometabolic risk profile: the international study of prediction of intra-abdominal adiposity and its relationship with cardiometabolic risk/intra-abdominal adiposity. Am J Clin Nutr. (2012) 96:714–26. 10.3945/ajcn.112.035758 22932278

[38] Anderson KM Castelli WP Levy D. Cholesterol and mortality 30 years of follow-up from the Framingham study. JAMA. (1987) 257:2176–80. 10.1001/jama.257.16.2176 3560398

[39] Castelli WP Anderson K. A population at risk. Prevalence of high cholesterol levels in hypertensive patients in the Framingham. Study Am J Med. (1986) 80:23–32. 10.1016/0002-9343(86)90157-9 3946458

[40] Gaziano JM Sesso HD Breslow JL Hennekens CH Buring JE. Relation between systemic hypertension and blood lipids on the risk of myocardial infarction. Am J Cardiol. (1999) 84:768–73. 10.1016/S0002-9149(99)00435-X 10513771

[41] Urbina EM Srinivasan SR Kieltyka RL Tang R Bond MG Chen W. Correlates of carotid artery stiffness in young adults: the bogalusa heart study. Atherosclerosis. (2004) 176:157–64. 10.1016/j.atherosclerosis.2004.04.023 15306189

[42] Selwyn AP Kinlay S Libby P Ganz P. Atherogenic lipids, vascular dysfunction, and clinical signs of ischemic heart disease. Circulation. (1997) 95:5–7. 10.1161/01.CIR.95.1.5 8994406

[43] Ruixing Y Jinzhen W Weixiong L Yuming C Dezhai Y Shangling P. The environmental and genetic evidence for the association of hyperlipidemia and hypertension. J Hypertens. (2009) 27:251–8. 10.1097/HJH.0b013e32831bc74d 19155782

[44] Egan BM. Insulin resistance and the sympathetic nervous system. Curr Hypertens Rep. (2003) 5:247–54. 10.1007/s11906-003-0028-7 12724058

